# Metabolic Signalling Peptides and Their Relation to Clinical and Demographic Characteristics in Acute and Recovered Females with Anorexia Nervosa

**DOI:** 10.3390/nu17081341

**Published:** 2025-04-14

**Authors:** Hiba Mutwalli, Johanna L. Keeler, Raymond Chung, Bethan Dalton, Olivia Patsalos, John Hodsoll, Ulrike Schmidt, Gerome Breen, Janet Treasure, Hubertus Himmerich

**Affiliations:** 1 Centre for Research in Eating and Weight Disorders (CREW), Department of Psychological Medicine, Institute of Psychiatry, Psychology and Neuroscience, King’s College London, London SE5 8AF, UKoliviapatsalos@gmail.com (O.P.); hubertus.himmerich@kcl.ac.uk (H.H.); 2 Department of Clinical Nutrition, College of Applied Medical Sciences, Imam Abdulrahman Bin Faisal University, Dammam 34221, Saudi Arabia; 3 NIHR BioResource Centre Maudsley, London WC2R 2LS, UK; 4 NIHR Maudsley Biomedical Research Centre (BRC), South London and Maudsley NHS Foundation Trust (SLaM), London SE5 8AF, UK; 5 Institute of Psychiatry, Psychology and Neuroscience, King’s College London, London SE5 8AB, UK; 6 Biostatistics & Health Informatics, Institute of Psychiatry, Psychology and Neuroscience, King’s College London, London SE5 8AF, UK; 7 Adult Eating Disorders Service, South London and Maudsley NHS Foundation Trust (SLaM), London SE6 4RU, UK; 8 Social, Genetic and Developmental Psychiatry Centre, Institute of Psychiatry, Psychology and Neuroscience, King’s College London, London SE5 8AF, UK

**Keywords:** markers, eating disorders, metabolic, biomarkers, depression, antidepressants

## Abstract

**Background/Objectives**: Recent research has established that metabolic factors may increase the vulnerability to develop anorexia nervosa (AN). The aim of this study was to explore the serum concentrations of leptin, insulin-like growth factor-1 (IGF-1), insulin and insulin receptor substrate (IRS-1) as possible state or trait biomarkers for AN in the acute and recovery (recAN) phases. Our secondary aim was to test associations between the tested markers and demographic and clinical characteristics. **Methods**: This cross-sectional study included data from 56 participants with AN, 24 recAN participants and 51 healthy controls (HCs). Enzyme-linked immunosorbent assays (ELISAs) were used to quantify serum concentrations of leptin, IGF-1, insulin and IRS-1. An analysis of covariance (ANCOVA) and linear regression models were utilised to test our results. **Results**: There were significant differences with a large effect size between the groups for serum leptin (*p* < 0.001; *d* = 0.80), whereby people with AN had lower leptin than those with recAN (*p* = 0.023; *d* = 0.35) and HCs (*p* < 0.001; *d* = 0.74). The between-group comparison of IGF-1 did not reach significance, although the effect size was moderate (*d* = 0.6) and was driven by lower levels of IGF-1 in people with acute AN compared to HCs (*p* = 0.036; *d* = 0.53). Serum insulin and IRS-1 did not differ between groups. **Conclusions**: Low leptin levels seen in individuals with AN may be due to starvation leading to fatty tissue depletion. Understanding the regulation of IGF-1 and insulin signalling over the course of the disorder requires further investigation.

## 1. Introduction

Recent research has established genetic correlations between anorexia nervosa (AN) and metabolic factors, including glycaemic, lipid and anthropometric traits [1], leading to suggestions that it could be reframed as a metabo-psychiatric disorder [2]. Therefore, metabolic factors may play a crucial role in the vulnerability to develop AN [3]. Metabolism in patients with AN slows down as a mechanism to preserve energy [4], and changes in metabolic signalling peptides may occur as an adaptive response to starvation. Several of these metabolic factors, in particular, leptin, insulin and insulin-like growth factor-1 (IGF-1), may constitute candidate biomarkers for AN [5].

Leptin is an adipocytokine involved in energy balance and metabolism [6]. It is produced by adipocytes within adipose tissues. It has a crucial role in signalling satiety as an appetite regulator [7]. Mutations in genes producing leptin cause early onset obesity [8,9]. Its role as a pharmacological agent for the treatment of lipodystrophy and for those with genetic defects has been well documented [10,11]. IGF-1 is a hormone with a similar molecular structure to insulin which is secreted by the liver and controlled by the pituitary growth hormone [12]. It is crucial for normal growth and plays an important role in anabolic processes, with the highest rates of production occurring during the pubertal period [13], which fall with aging [14]. IGF-1 has a role in sustaining cell growth in almost all body systems [15]. Insulin, produced by beta cells of the pancreatic islets, is the main regulatory hormone that controls glucose homeostasis [16]. In the post-absorptive phase, it suppresses glucose production, enhances glucose reuptake from the liver and muscles and downregulates lipolysis [16]. IRS-1 is a substrate of the insulin and IGF-1 receptors [17]. It plays a crucial role in insulin signalling pathways, which is in turn necessary for glucose homeostasis [18]. IRS-1 plays a substantial role in the transmission of signals from both the receptor for insulin and IGF-1 [19,20], in addition to its biological role in metabolism, cell division and growth [21].

Body mass index (BMI) and body fat impact on the levels of metabolic peptides, including leptin, IGF-1, insulin and IRS-1 [22]. For example, leptin directly relates to BMI and body fat due to it being produced from adipose tissues [23]. Insulin promotes fat production and storage by regulating lipid metabolism (e.g., lipogenesis and lipolysis) in adipose tissues [24]. An increase in body weight or fat (especially visceral fat) leads to an increase in insulin production and a reduction in insulin sensitivity [25]. IGF-1 and IRS-1, are involved in this process [26,27].

In AN, however, where the volume of adipose tissue is reduced, evidence from meta-analyses shows significantly lower leptin and insulin in AN than HCs [5,28]. Patients with AN show resistance to growth hormone, which leads to lower release of IGF-1 [29,30,31,32]. Other growth factors such as vascular endothelial growth factor, brain-derived neurotrophic factor and IGF-1 are reduced in AN [33,34]. To the best of our knowledge, no studies have investigated IRS-1 in AN, despite it being considered a crucial component in the insulin signalling pathway [35]. IRS-1 is also involved in fat and muscle metabolism processes [36]. IRS-1 disturbance has been linked with insulin sensitivity, which is found to be increased in individuals with AN [37,38]. In addition, as IRS-1 reflects the current insulin action in the body, it may be useful as a monitoring biomarker. Understanding how insulin signalling pathways are affected in individuals with AN might reveal the intracellular metabolic adaptation to starvation.

The aim of the current study is to examine peripheral concentrations of appetite regulators and metabolic and growth factors in the acute state and after long-term recovery. We hypothesised that leptin, IGF-1 and insulin are lower in AN in response to weight loss and therefore normalise after recovery. As a secondary investigation, we hypothesised that leptin is positively associated with BMI and levels of body fat, and leptin and IGF-1 are associated with eating disorder psychopathology. Finally, as an exploratory analysis, we aimed to test the association between biological markers and depression diagnosis.

## 2. Materials and Methods

### 2.1. Participants and Recruitment

**Figure 1:**
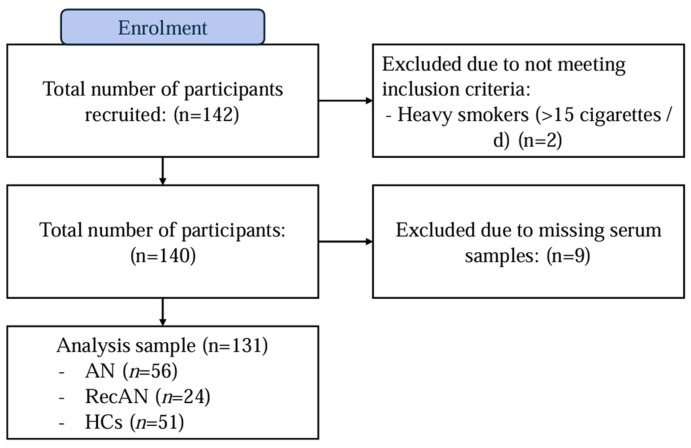
Flow diagram of participant recruitment. Abbreviations: AN = anorexia nervosa; recAN = recovered anorexia nervosa and HCs = healthy controls.

This study employed a cross-sectional design to compare biological samples from 131 adult female participants (≥18 years old) included in a previous study [39,40]. A total of 142 participants were originally recruited for this study, but two participants were excluded due to not meeting inclusion criteria, and nine were excluded due to missing serum blood samples (Figure 1). The final sample included participants with acute AN (AN; *n* = 56), people who had recovered from AN (recAN; *n* = 24) and healthy controls (HCs; *n* = 51). The AN and recAN groups were recruited from both clinical settings and previous study registries. HCs were recruited through online and community advertisements.

A screening call was conducted to confirm the suitability of participants to take part in the study. Exclusion criteria for all participants were as follows: male, under 18 years of age, pregnant, experiencing any condition that may affect biological values (e.g., the presence of inflammatory conditions), current abuse of alcohol (≥3 units a day, 5 days a week on average) and heavy smoking (≥15 cigarettes a day). The AN diagnosis was ascertained according to the Diagnostic and Statistical Manual for Mental Disorders (DSM-5) [41] using the Eating Disorder Diagnostic Scale [42]. Participants in the AN group were included if they had a current AN diagnosis according to DSM-5 and a BMI of ≤18.5 kg/m^2^. RecAN participants were recruited if they had a previous AN diagnosis meeting the DSM-5 criteria but had an absence of eating disorders behaviours and had maintained a BMI ≥ 18.5 kg/m^2^ for at least 6 months prior to participation. HCs were recruited if they had no current or past psychiatric disorder as confirmed by the Structured Clinical Interview for DSM-5 [41]. All participants consented to take part in the study.

Participants who were eligible and consented to take part in the study visited King’s College London (KCL), where they completed a demographic and clinical assessment. The assessment session involved completing self-reported questionnaires (e.g., demographics, clinical, pharmaceutical and anthropometrics data). Comprehensive body composition and anthropometric measurements were obtained using the InBody S10 research grade body composition analyser, the results of which are published elsewhere [40]. Serum blood samples (5 mL) were collected from participants by a trained lab technician. For more details about the study design, inclusion/exclusion criteria and the sample size calculation, refer to [39,40].

### 2.2. Measures

#### 2.2.1. Self-Report Questionnaires

##### Demographic and Clinical History

Participants completed a bespoke questionnaire measuring variables relating to their demographics and clinical history. Of these, age and psychotropic medication usage were used in this study.

##### Body Mass Index

Participants’ weight and height was measured, and BMI was calculated as body weight [kg]/height^2^ [m^2^].

##### Eating Disorder Psychopathology

The Eating Disorder Examination Questionnaire (EDE-Q) Version 6.0 (28 items) [43] was used to assess the symptoms of eating disorders over the previous 28 days. The questionnaire produces a total score and four subscale scores, including restrained eating, eating concern, shape concern and weight concern.

##### Presence of Depression and Severity of Depressive Symptoms

The Beck Depression Inventory-II [44], a self-reported questionnaire consisting of 21 questions relating to depression symptoms, was utilised to measure severity of depressive symptoms. Additionally, participants were asked to report whether they had a lifetime diagnosis of a depressive disorder (or any other psychiatric disorder) in the demographic and clinical characteristics questionnaire.

#### 2.2.2. Metabolic Signalling Peptide Measurement

Blood samples were collected from participants in two 10 mL ethylenediaminetetraacetic acid (EDTA) tubes and a 5 mL serum tube. Serum samples were separated with centrifuging and stored at −80 °C in the laboratories of the National Institute for Health and Care Research, Biomedical Research Centre (NIHR BRC) BioResource. Samples were processed by experienced staff at the Social, Genetic and Developmental Psychiatry Centre (SGDP) at King’s College London. Prior to processing, samples were thawed at room temperature. Electrochemiluminescence (ECL) assays were used to quantify the concentrations of blood leptin, IGF-1 and IRS-1 in pg/mL and blood insulin in uIU/mL using commercially available kits manufactured by Meso Scale Discovery (Meso Scale Discovery, Rockville, MD, USA). The U-PLEX metabolic 2-Plex combo 1 (hu) sector (1 PL) was used to measure human leptin and insulin. The R-PLEX human IRS-1 assay and IGF-1 assay sector (5 PL) were used to measure IRS-1 and IGF-1 concentrations. The concentrations of leptin, IGF-1, insulin and IRS-1 were quantified following the manufacturer’s instructions. IGF-1 and IRS-1 were quantified in duplicate, and mean values of these duplicates are reported. Table S1 details the lower and upper detection limits according to the NIHR BRC BioResource laboratories. All groups were randomised across the analysis batches and plates. Although measurements were processed separately in two different analysis assay plates (Table S2), they were measured in a single point; therefore, no intraassay CVs are presented. No missing biological values were found in our data.

#### 2.2.3. Statistical Analysis

All statistical analyses were conducted using SPSS 29.0.2.0 [45] and R version 4.3.1 [46]. Figures were produced using the “ggplot2” and “patchwork” packages in R. Values throughout the paper are expressed as means and standard deviations (SDs) or median and interquartile range where data were non-normally distributed. The normality of the data distribution for continuous variables was assessed using the Shapiro–Wilk test and violin plots (Figure S1), whereas the distribution of categorical variables was assessed using the chi-square goodness-of-fit test. Non-normally distributed variables were log10-transformed prior to inclusion in the main statistical models. Results were considered significant when the *p*-values were equal to or less than 0.05. Cohen’s *d* values of 0.2, 0.5 and 0.8 were interpreted as small, moderate and large effect sizes, respectively. The power analysis of this cohort was previously assessed using a priori power analysis using one-way analysis of covariance (ANCOVA) with independent samples, which was conducted using the means and standard deviations of a previous study [33]. The power analysis revealed significant findings (effect size = 0.8; power level = 0.8, *p* = 0.00135). Our sample exceeded the required number to detect the effect with a proposed number of 17 per group, which was determined to be sufficient.

Based on the literature, age and cigarette smoking are associated with alterations in several of these markers; for example, leptin and IGF-1 levels were found to be lower in smokers compared to non-smokers, while insulin resistance was higher in smokers [47,48,49,50], and older age is associated with an increase in both leptin and insulin resistance [51,52]. Therefore, these variables were controlled for in all analyses (i.e., age (years) and smoking (yes/no)).

##### Comparisons of Demographic and Clinical Characteristics Between Groups

Demographic and clinical characteristics were compared between groups using one-way analyses of variance (ANOVAs) with Tukey’s post hoc tests [53] for pairwise comparisons (continuous variables) and chi-square tests (categorical variables).

##### Cross-Sectional Comparisons of Metabolic Signalling Peptides Between Groups

To compare concentrations of leptin, IGF-1, insulin and IRS-1 between the AN group, recAN group and HCs, analyses of covariance (ANCOVAs) was used, controlling for age (years) and smoking (yes/no), with Tukey’s post hoc tests to assess pairwise comparisons. We measured the differences in biological concentrations between the two measurement plates (i.e., plate 1 and 2) using parametric analysis of variance (ANOVA) and adjusted main analyses for the plate number where differences were significant. As a sensitivity analysis, we further adjusted for the use of psychotropic medications when measuring the differences in biological markers in groups using ANCOVAs.

##### Exploratory Linear Regression Analysis Between Metabolic Signalling Peptides and Demographic and Clinical Characteristics

Linear regression analyses assessed associations within the AN group (*n* = 56) between levels of leptin, insulin, IGF-1 and IRS-1 as dependent variables, with BMI, body fat percentage, depression diagnosis and EDE-Q global scores and subscales (e.g., restrained eating, eating concerns, shape concerns and weight concerns) as regressors, tested separately in each analysis model. Age and smoking status were included as covariate regressors in all analysis models. Standardised and unstandardised beta coefficients with 95% confidence intervals are presented. These regression models were also repeated in participants with acute AN and recAN combined (*n* = 80).

## 3. Results

### 3.1. Descriptive Statistics of the Sample

Age was different between groups, whereby participants in the AN group were significantly older than HCs. As expected, participants with AN had a lower BMI and higher EDE-Q scores than HCs. The recAN group did not differ in BMI from HCs but had higher EDE-Q scores (albeit lower than in the acute AN group). The restrictive subtype of AN was more dominant than the binge–purge, with 84% of the AN and 100% of the recAN reporting an AN-R subtype (Table 1).

### 3.2. Differences in Biological Markers Between Group

No missing or undetectable biological values were found in our data. The descriptive statistics of the metabolic signalling peptides are presented in Table 2. A number of extreme outliers (*n* = 30) were detected. Additionally, comparisons of the biological markers across analysis plates showed differences in IRS-1 concentrations but no differences in the other markers (Table S3). Therefore, the analysis of IRS-1 was additionally adjusted for the plate number.

**Figure 2:**
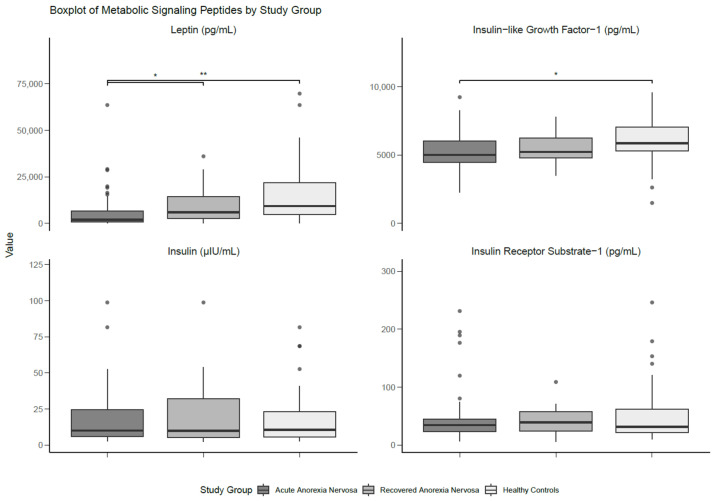
Boxplot of metabolic signalling peptides differentiated by study group. Medians and interquartile ranges (IQRs) are shown. Error bars represent the smallest and the largest values within 1.5 of the IQR. * Significant at *p* < 0.05. ** Significant at *p* < 0.01.

There were significant differences with a large effect size between the groups for serum leptin (*p* < 0.001; *d* = 0.80), whereby individuals with AN had lower levels than those with recAN (*p* = 0.023; *d* = 0.35) and HCs (*p* < 0.001; *d* = 0.74). No statistical differences in leptin levels were found between those with recAN and HCs. Serum IGF-1 was not significantly different between groups, although the analysis yielded a moderate effect size (*d* = 0.60). Pairwise comparisons showed significantly lower levels of IGF-1 in participants with AN compared to HCs, with a moderate effect size (*p* = 0.036; *d* = 0.53). There were no significant findings for either serum insulin or IRS-1. The results of the between-group comparisons are presented in Table 3 and Figure 2.

After adjusting for the use of psychotropic medications and antidepressant use independently, leptin remained significantly different between groups (model adjusted for age, smoking and psychotropic medication use: *p* < 0.001; *d* = 0.88; model adjusted for age, smoking and antidepressant use: *p* < 0.001; *d* = 0.81), and the pairwise comparisons likewise remained significant. However, after adjusting for antidepressant or antipsychotic usage, differences in concentrations of IGF-1 between participants with AN and HCs were not found (Tables S4 and S5).

### 3.3. Exploratory Linear Regression Analysis of the Associations Between Clinical and Demographic Characteristics and Metabolic Signalling Peptides in Anorexia Nervosa (n = 56) and in Acute and Recovered Anorexia Nervosa Combined (n = 80)

In the group of individuals with acute AN (*n* = 56), serum leptin was found to be positively associated with BMI (*β* = 0.44; *p* < 0.001; *d* = 1.11) and the percentage of body fat (*β* = 0.53; *p* < 0.001; *d* = 1.32), with large effect sizes. BMI also had a moderate-sized but non-significant positive association with insulin (*β* = 0.20; *d* = 0.52). There was a positive association between blood IGF-1 and the presence of a depression diagnosis, with a moderate effect size (*β* = 0.26; *p* = 0.036; *d* = 0.66). No associations were seen in any of the other tested markers or measures (e.g., the EDE-Q) (Table 4, Tables S6 and S7).

The above associations were also found when combining the AN and recAN sample. However, in this larger combined sample, a negative association between serum leptin and the global score (*β* = −0.24; *p* = 0.043; *d* = −0.50) and eating concern subscale (*β* = −0.27; *p* = 0.016; *d* = −0.57) of the EDE-Q was found. No other significant associations were found for insulin and IRS-1 (Table 4, Tables S8 and S9).

## 4. Discussion

The aim of this paper was to compare the profile of leptin, IGF-1, insulin and the substrate of the insulin receptor, IRS-1, in the acute and recovered stages of AN with HCs. We also examined associations between these biological markers and key demographic and clinical characteristics, including BMI, body fat, eating disorder psychopathology, the history of depression diagnosis and the use of psychotropic medications. Overall, we found reductions in peripheral concentrations of leptin and IGF-1 in the acute stages of AN, compared with HCs. Contrary to the hypothesis, peripheral concentrations of insulin and IRS-1 did not differ between groups. As hypothesised, leptin was negatively associated with both BMI and body fat percentage in the AN sample and AN and recAN combined sample. Eating disorder psychopathology and in particular, the eating concern subscale were negatively associated with leptin concentrations but only in the combined sample of acute and recovered AN individuals. Finally, it was found that IGF-1 concentrations were higher in people with AN or recAN that reported a history of depression.

In line with previous studies [5,28], individuals with acute AN had lower leptin levels compared to those with recAN and HCs, even when controlling for the use of psychotropic medication. We also found a modest difference between individuals with AN and HCs in IGF-1 levels, which is in line with previous investigations [28,34]. The noted differences in IGF-1 between participants with AN and HCs were diminished after adjusting for the use of psychotropic medications. Our findings regarding insulin were inconsistent with recent meta-analytical findings that noted lower insulin levels in acute AN [5]. It is likely that this is related to a lack of standardisation across participants in terms of meal timing and fasting state. The lack of difference in serum IRS-1 between the groups is potentially due to compensatory regulatory mechanisms in insulin signalling based on nutritional status (i.e., efforts to maintain homeostasis despite metabolic stress), which may prevent variations across groups [54,55].

We found a strong association between leptin and BMI and body fat in the acute and recovered AN individuals and the combined group. The association noted between serum leptin and eating disorder psychopathology supports recent arguments that hypoleptinaemia is a trigger not solely for the body size but also brain functioning [56]. Previous research similarly found a negative association between leptin and eating disorder psychopathology [57], which may be related to the role of leptin in reward circuits [58]. However, as the change was noticed solely in the combined group of acute and recovered AN individuals and not in the acute AN group alone, this may be attributed to the influence of metabolic adaptation and weight restoration on leptin that made the relationship more apparent in the combined group. For example, using a more diverse sample with wider body weight range may have made the linearity of the relationship between levels of leptin and eating disorder psychopathology more noticeable. However, the absence of a relationship in the acute AN group may reflect individuals falling below a threshold in which they are hypoleptinaemic [56] or may be due to lower statistical power. The association between IGF-1 and depression in the acute and recovered AN phases is consistent with recent meta-analytical evidence which showed increased circulatory IGF-1 in patients with depressive disorder compared to HCs [59,60].

### 4.1. Clinical Implications

Leptin levels may give us an insight into the current nutritional status and recovery progress of a person with AN. Although studies have conflicting findings, with some finding that higher leptin levels are associated with reduced likelihood of relapse [61], while others show no relation between leptin levels and long-term prediction of weight recovery [62], it may be worthwhile to investigate its relation to psychological recovery. Prolonged downregulation of leptin has been shown to be associated with several body systems, and a threshold for hypoleptinaemia of 4 ng/mL has been hypothesised, under which “entrapment” in the eating disorder may be triggered [56]. Moreover, hypoleptinaemia may influence changes in many other biological systems, including metabolism, immunity and reproduction, among others. For example, in addition to its role in regulating metabolism and appetite, it is also a key regulator for the immune, reproductive, growth, neuroendocrine and autonomic systems and bone metabolism [63,64,65,66]. Therefore, it is not solely an indicator of physiological changes in acute AN individuals but may also give an insight into further systematic recovery. In addition, leptin levels may trigger the reward-related behaviours and psychological processes which play a role in the maintenance of eating disorder symptomatology [57]. It has been hypothesised that higher serum leptin levels in acutely unwell patients with AN may be linked with a faster improvement in AN psychopathology [56,67], thus constituting a potential predictive biomarker, although this needs further empirical exploration.

The association of IGF-1 with depression could indicate its usefulness as a marker for psychological improvement during the course of treatment of AN, considering its role in neuroplasticity, cognition and mood regulation [59,68]. Although this association is controversial, recent meta-analytical evidence showed an increase in IGF-1 in patients with depression [60] and that it might be utilised as a trade marker for depression [69]. This correlation requires further in-depth understanding, especially considering that it involves many pathways such as the growth hormone-IGF-1 axis, metabolic adaptation (i.e., leptin, insulin and ghrelin) and hypothalamic-pituitary adrenal axis [68,70,71,72].

Additionally, research has started to explore the role of the gut microbiota in AN [73]. Although research has considered the metabolic underpinning, it has shown that the gut microbiota influences the individual’s metabolism, in addition to immunity and the central nervous system, through the gut–brain axis [74,75,76]. Gut dysbiosis seen in individuals with AN affects the metabolites, inflammatory system and the synthesis of neurotransmitters [77,78]. Therefore, investigating the role of the gut microbiota together with the metabolic processes may provide valuable insight into the aetiology of AN.

Collectively, this evidence sheds light on new therapeutic interventions, which may include dietary supplements [79] in addition to conventional AN treatments which targets both the nutritional needs and the gut–brain interaction. For example, probiotics has shown to positively affect the microbiota and therefore positively enhance weight gain and reduce gastrointestinal symptoms [73]. Omega-3 fatty acids and fermented foods have also shown some promising characteristics, especially those related to their effect on the gut microbiota and the subsequent improvement in outcomes such as the psychological behaviours of AN [80].

### 4.2. Strengths and Limitations

To the best of our knowledge, there are no studies that have explored the intracellular markers in AN, and therefore the investigation of IRS-1 is a novel aspect of the study. Previous studies on patients with Alzheimer’s disease suggested that dysfunction in IRS-1 may play a role in cognitive decline [81]. Our study indicates that alterations in IRS-1 are not associated with AN in the acute or recovered state.

However, the findings of the present study should be interpreted in light of several important limitations. Firstly, the influence of potential confounders could not be fully accounted for (i.e., fasting state, meal timings and medication use). Detailed information about the duration of use of psychotropic medications was not available. Another limitation of this study is related to the sample size of the recAN group, which may have potentially reduced the statistical power, particularly for the between-group comparisons of the biological values. In addition, the cross-sectional nature of this investigation limited our ability to draw conclusions about the directionality of the relationship in our regression analyses.

### 4.3. Future Directions

Considering the role of IRS-1 in insulin and IGF-1 signalling [19,82] and the limitations of the current study, it may be worthwhile to replicate the investigations conducted on IRS-1 while accounting for possible confounders, including the fasting state, meal timings and medication use. Additionally, examining the roles of insulin and IRS-1 longitudinally in the partial and full weight restoration phases and long-term recovery phases of AN may provide additional knowledge about the underlying metabolic and physiological mechanisms, which may act as predictors for recovery. Lastly, the association between IGF-1 and depressive comorbidity in AN populations may be further explored in regard to its role in neuroplastic processes. Future research investigating other promising markers for AN may be of interest. For example, studies investigating the role of adipokines such as adiponectin, omentin and visfatin may provide an insight into the aetiology of AN. Previous research has constantly shown that adiponectin is higher in AN individuals and was shown to be higher in both AN subtypes (i.e., AN-R and AN-B/P) [83,84]. However, the roles of omentin and visfatin are less clear but show promising results, especially those relating to the link of visfatin with obsessive and compulsive symptoms and the non-homeostatic regulation of eating, while omentin is shown to be elevated in AN, suggesting a unique regulatory mechanism in response to starvation (in the acute phase because it does not normalise after recovery) [85].

Adiponectin can be modified by insulin, IGF-1 and BMI, which indicates its interaction with metabolic parameters [83].

## 5. Conclusions

In this study, we found significant differences in leptin levels among the groups, corroborating previous findings of hypoleptinaemia in AN. IGF-1 was lower in people with AN compared with HCs but higher in participants with AN who had a history of depression, indicating that IGF-1 may play a role in affective comorbidity in people with AN. For the first time, IRS-1 was investigated in this population, although there were no differences between groups. Concentrations of insulin were also not different between groups.

## Figures and Tables

**Table 1:** Descriptive statistics of the study sample.

	AN (*n* = 56)	RecAN (*n* = 24)	HCs (*n* = 51)	*p*-Value (Cohen’s *d*)			
				Total Model	AN vs. HC	AN vs. recAN	RecAN vs. HC
Demographic characteristics							
Age (years) (mean ± SD)	26.59 ± 8.13	26.58 ± 6.14	23.53 ± 3.92	0.032 * (0.47)	0.040 * (−0.47)	1.000 (−0.001)	0.137 (**−0.65**)
BMI (kg/m^2^) (mean ± SD)	15.94 ± 1.27	20.7 ± 1.94	21.22 ± 1.81	<0.001 *** (3.15)	<0.001 *** (3.40)	<0.001 *** (3.17)	0.404 (0.28)
Body fat % (mean ± SD)	11.73 ± 4.94	22.35 ± 6.15	24.01 ± 5.11	<0.001 *** (2.25)	<0.001 *** (2.44)	<0.001 *** (1.90)	0.413 (0.29)
Clinical characteristics							
AN subtype, n (%)							
AN-R	47 (83.93)	24 (100)	0	-	-	0.037 * (0.51)	-
AN-B/P	9 (16.07)	0	0	-	-	-	-
Depression diagnosis n (%)	18 (10.08)	4 (16.67)	0	-	-	0.114 (**0.50**)	-
BDI (mean ± SD)	30.55 ± 11.43	11.63 ± 9.47	3.98 ± 4.54	<0.001 *** (2.97)	<0.001 *** (−3.00)	<0.001 *** (−1.74)	0.002 ** (−1.18)
Antidepressant use n (%)	26 (46.4)	6 (25)	0	-	-	0.073 (0.44)	-
Antipsychotics n (%)	6 (10.7)	2 (8.3)	0	-	-	0.061 (**0.55**)	-
Eating disorder psychopathology							
Global ^a^ (mean ± SD)	3.9 ± 1.15	1.01 ± 0.84	0.56 ± 0.64	<0.001 *** (3.02)	<0.001 *** (3.07)	<0.001 *** (2.18)	0.007 ** (0.66)
Eating ^a^ restraint (mean ± SD)	3.76 ± 1.31	0.68 ± 0.96	0.55 ± 0.81	<0.001 *** (2.67)	<0.001 *** (2.75)	<0.001 *** (2.1)	0.944 (0.02)
Eating ^a^ concern (mean ± SD)	3.54 ± 1.31	0.73 ± 0.85	0.14 ± 0.23	<0.001 *** (3.52)	<0.001 *** (3.33)	<0.001 *** (2.08)	<0.001 *** (1.1)
Shape concern ^a^ (mean ± SD)	4.46 ± 1.28	1.52 ± 1.1	0.9 ± 0.91	<0.001 *** (2.76)	<0.001 *** (2.84)	<0.001 *** (1.95)	0.004 ** (0.71)
Weight concern ^a^ (mean ± SD)	3.85 ± 1.45	1.1 ± 0.88	0.65 ± 0.84	<0.001 *** (2.54)	<0.001 *** (2.65)	<0.001 *** (1.84)	0.017 * (0.57)

^a^ Log-transformed. Parametric one-way analyses of variance (ANOVAs) were used; * significant at *p* < 0.05; ** significant at *p* < 0.01; *** significant at *p* < 0.001; effect size represented as Cohen’s *d* of 0.2, 0.5 and 0.8 for small, moderate and large effect sizes, respectively; moderate and large values of *d* are emboldened. Abbreviations: AN = anorexia nervosa; recAN = recovered anorexia nervosa; HCs = healthy controls; AN-R = anorexia nervosa—restricting; AN-B/P = anorexia nervosa—binging/purging; BDI = Beck’s depression inventory.

**Table 2:** Descriptive statistics of the metabolic signalling peptides represented as median and interquartile range.

Metabolic Signalling Peptides		AN (*n* = 56) *	AN-R (*n* = 47)	AN-B/P (*n* = 9)	recAN (*n* = 24)	HCs (*n* = 51)
Leptin (pg/mL)	Median (IQR)	1885.9 (5922.6)	1977.9 (5888.36)	1470.73 (11,488.74)	5855.11 (12,194.68)	9302.18 (17,462.16)
	Mean ± SD	5914.98 ± 10,441.88	5062.29 ± 7092.73	10,367.91 ± 20826.86	9527.08 ± 9902.68	15,642.09 ± 15,547.6
IGF-1 (pg/mL)	Median (IQR)	5017.19 (1605.15)	5033.89 (1626.23)	4550.79 (1919.65)	5229.78 (1765.28)	5870.89 (1813.47)
	Mean ± SD	5164.1 ± 1379.96	5197.27 ± 1417.71	4990.88 ± 1222.61	5520.14 ± 1151.79	5954.94 ± 1588.81
Insulin (µIU/mL)	Median (IQR)	10.13 (19.04)	11.07 (20.5)	8.6 (12.1)	9.88 (29.73)	10.69 (17.62)
	Mean ± SD	17.59 ± 18.73	18.94 ± 19.89	10.52 ± 8.46	21.4 ± 23.64	17.9 ± 18.14
IRS-1 (pg/mL)	Median (IQR)	34.68 (22.22)	35.04 (24.79)	30.78 (14.97)	39.25 (38.03)	31.49 (41.6)
	Mean ± SD	46.58 ± 46.83	49.19 ± 50.49	32.91 ± 12.91	42.13 ± 24.68	48.23 ± 46.53

Note: * Median values should be used as a representation of the group average as there was considerable left skewness in the data. Abbreviations: AN = anorexia nervosa; AN-B/P = anorexia nervosa—binging/purging; AN-R = anorexia nervosa—restricting; recAN = recovered anorexia nervosa; HCs = healthy controls; IGF-1 = insulin-like growth factor-1; IRS-1 = insulin receptor substrate-1.

**Table 3:** Differences in the metabolic signalling peptides between groups and pairwise, adjusted for age and smoking.

Metabolic Signalling Peptides	Adjusted M ± SE			*p* (Cohen’s *d*)	*F*	*df*	*p* (Cohen’s *d*)		
	AN (*n* = 56)	recAN (*n* = 24)	HCs (*n* = 51)	Total Model			AN vs. HC	AN vs. RecAN	RecAN vs. HC
Leptin ^a^ (pg/mL)	3.28 ± 0.09	3.65 ± 0.13	3.93 ± 0.09	<0.001 ** (0.80)	12.39	2, 126	<0.001 ** (0.74)	0.023 * (0.35)	0.098 (0.44)
IGF-1 (pg/mL)	5249.21 ± 184.58	5600.44 ± 280.55	5823.69 ± 195.19	0.110 (**0.60**)	2.28	2, 126	0.036 * (0.53)	0.295 (0.27)	0.518 (0.30)
Insulin ^a^ (µIU/mL)	1.08 ± 0.05	1.11 ± 0.08	1.07 ± 0.06	0.899 (0.20)	0.11	2, 126	0.932 (0.02)	0.697 (0.19)	0.655 (−0.18)
IRS-1 ^ab^ (pg/mL)	1.55 ± 0.04	1.55 ± 0.06	1.53 ± 0.04	0.363 (0.26)	1.02	2, 125	0.82 (0.06)	0.372 (0.21)	0.155 (0.35)

^a^ Log-transformed; ^b^ IRS-1 was additionally controlled for the plate number; results based on analysis of covariance (ANCOVA) models; significant *p*-values are presented in bold; * significant at *p* < 0.05; ** significant at *p* < 0.01. Cohen’s *d* values of 0.2, 0.5 and 0.8 represent small, moderate and large effect size; moderate and large effect sizes are also denoted in bold. Abbreviations: Adjusted M ± SE = adjusted mean ± standard error; AN = anorexia nervosa; HCs = healthy controls; IGF-1 = insulin-like growth factor-1; IRS-1 = insulin receptor substrate-1; recAN = recovered anorexia nervosa.

**Table 4:** Association between clinical and demographic characteristics and the metabolic signalling peptides in the AN group (*n* = 56) and acute and recovered AN groups combined (*n* = 80), controlling for age and smoking.

Metabolic Signalling Peptides	Body Mass Index		% Body Fat		EDE-Q Global Score ^a^		Depression Diagnosis	
	*β*	*p* (*d*)	*β*	*p* (*d*)	*β*	*p* (*d*)	*β*	*p* (*d*)
Acute AN (*n* = 56)								
Leptin ^a^, (pg/mL)	0.44	<0.001 ** (1.11)	0.53	<0.001 ** (1.32)	0.09	0.513 (0.29)	0.14	0.337 (0.38)
IGF-1, (pg/mL)	0.15	0.246 (0.40)	0.11	0.380 (0.33)	−0.01	0.925 (−0.02)	0.26	0.036 * (0.66)
Insulin ^a^, (µIU/mL)	0.20	0.139 **(0.52)**	0.00	0.980 (0.11)	−0.17	0.238 (−0.34)	−0.07	0.612 (−0.14)
IRS-1 ^a^, (pg/mL)	−0.22	0.090 (−0.46)	−0.14	0.280 (−0.29)	−0.18	0.187 (−0.36)	0.03	0.805 (0.17)
Acute and recovered AN (*n* = 80)								
Leptin ^a^, (pg/mL)	0.37	<0.001 ** (0.92)	0.45	<0.001 ** (1.16)	−0.24	0.043 * (−0.50)	0.06	0.636 (0.21)
IGF-1, (pg/mL)	0.15	0.164 (0.40)	0.18	0.096 (0.46)	−0.10	0.370 (−0.20)	0.23	0.031 * (0.58)
Insulin ^a^, (µIU/mL)	0.18	0.118 **(0.50)**	0.14	0.213 (0.39)	−0.14	0.236 (−0.28)	−0.02	0.843 (−0.05)
IRS-1 ^a^, (pg/mL)	−0.08	0.455 (−0.17)	−0.01	0.958 (−0.01)	−0.08	0.470 (−0.17)	0.03	0.811 (0.16)

^a^ Log-transformed. Significance level at the threshold of * *p* < 0.05, ** *p* < 0.01; *d* values of 0.2, 0.5 and 0.8 represent small, moderate and large effect sizes. Emboldened Cohen’s *d* represents a moderate or large effect size. Abbreviations: *β* = standardised beta coefficient; *d* = Cohen’s effect size; EDE-Q = Eating Disorder Examination Questionnaire; IGF-1 = insulin-like growth factor-1; IRS-1 = insulin receptor substrate-1.

## Data Availability

The original contributions presented in this study are included in the article/Supplementary Materials. Further inquiries can be directed to the corresponding author.

## Supplementary Materials

The following supporting information can be downloaded at: https://www.mdpi.com/article/10.3390/nu17081341/s1, Table S1: Metrics of the metabolic signalling peptides in the whole sample; Table S2. Distribution of participants’ serum samples into analysis plates; Table S3. Differences in biological values between analysis plates 1 and 2 in the whole analysis sample (*n* = 131); Table S4. Differences in the metabolic signalling peptides between groups, adjusting for age, smoking and psychotropic medication use; Table S5. Differences in the metabolic signalling peptides between groups, adjusting for age, smoking and antidepressant use; Table S6. Differences in metabolic signalling peptides in acute AN (*n* = 56) separated by the presence or absence of a lifetime depression diagnosis, controlling for age and smoking; Table S7. Differences in metabolic signalling peptides in acute and recovered AN individuals (*n* = 80) separated by the presence or absence of lifetime depression diagnosis, controlling for age and smoking; Table S8. Association between clinical and demographic characteristics with the metabolic signalling peptides in the acute and recovered anorexia nervosa group (*n* = 80) controlling for age and smoking; Table S9. Association between eating disorder psychopathology and the metabolic signalling peptides in the acute and recovered anorexia nervosa group (*n* = 80), controlling for age and smoking; Figure S1. Boxplot of insulin-like growth factor-1 in AN (*n* = 56) differentiated by the presence or absence of lifetime depression diagnosis.

## Abbreviations

The following abbreviations are used in this manuscript (listed according to appearance in the text):

AN	Acute anorexia nervosa
IGF-1	Insulin-like growth factor-1
IRS-1	Insulin receptor substrate
RecAN	Recovered anorexia nervosa
HC	Healthy control
ECL	Electrochemiluminescence
ANCOVA	Analysis of covariance
BMI	Body mass index
DSM-5	Diagnostic and Statistical Manual for Mental Disorders-5
KCL	King’s College London
EDE-Q	Eating Disorder Examination Questionnaire
NIHR	National institute for health and care research
BRC	Biomedical research centre
SGDP	Social, genetic and developmental psychiatry centre
SD	Standard deviation
ANOVA	Analysis of variance
n	Number
*d*	Cohen’s *d*
AN-R	Anorexia nervosa restricting type
AN-B/P	Anorexia nervosa binge/purge type
BDI-II	Beck Depression Inventory-II
IQR	Interquartile range
Pg/mL	Picograms per millilitre
µIU/mL	Micro international units per millilitre
*β*	Beta coefficient
SLaM	South London and Maudsley
