# Supplementary material for: Metabolic Signalling Peptides and Their Relation to Clinical and Demographic Characteristics in Acute and Recovered Females with Anorexia Nervosa
Source: Nutrients. 2025 Apr 14;17(8):1341. doi: 10.3390/nu17081341 (PMC12030328; doi:10.3390/nu17081341)
Supplement: Supplementary file 1 [file nutrients-17-01341-s001.zip › nutrients-3536384-supplementary.pdf]

Metabolic Signalling Peptides and Their Relation to Clinical and Demographic Characteristics in Acute and Recovered Females with Anorexia Nervosa

Hiba Mutwalli, Johanna L. Keeler, Raymond Chung, Bethan Dalton, Olivia Patsalos, John Hodsoll, Ulrike Schmidt, Gerome Breen, Janet Treasure and Hubertus Himmerich

Supplementary materials

1. **Supplementary tables**

Table S1. Metrics of the metabolic signaling peptides in the whole sample.

Metabolic signalling biomarker	Low detection limit		High detection limit	
	Plate 1	Plate 2	Plate 1	Plate 2
**Leptin (pg/mL)**	16.1	16.1	49700	49700
**IGF-1 (pg/mL)**	565	690	40000	40000
**Insulin (μIU/mL)**	0.0799	0.0799	686	686
**IRS-1 (pg/mL)**	13.7	10.5	200000	200000

Detection limits were reported by NIHR BioResource. All values were detectable and no below detection limits values found in this sample.

Table S2. Distribution of participants serum sample into analysis plates.

	AN (n=56)	RecAN (n=24)	HC (n=51)	*P*-value (Cohens *d*)
**Plate n (%)**				
**1**	40 (71.43)	7 (29.2)	33 (64.71)	**<0.001*** (0.67)**
**2**	16 (29.1)	17 (70.8)	18 (34.6)	

P-value was assessed using the Pearson Chi Square test; Effect size (Cohens *d*) was obtained from the Chi Square test value; ***Significant at threshold of $p<0.001$; Cohen *d* classified as small ($d=0.2$), moderate ($d=0.5$) or large ($d=0.8$); Significant *p* values and moderate or large Cohen *d* are emboldened. Abbreviations: AN = anorexia nervosa; RecAN = recovered anorexia nervosa; HC = healthy controls.

Table S3. Differences in biological values between analysis plates 1 and 2 in the whole analysis sample (n=131).

**Metabolic signalling peptides**	**1 (n=80)**	**2 (n=51)**	***p*-value**
**Leptin[a] (pg/mL)**	3.59 ± 0.72	3.62 ± 0.70	0.771
**IGF-1 (pg/mL)**	5660.87 ± 1574.88	5343.25 ± 1257.66	0.227
**Insulin[a] (µIU/mL)**	1.06 ± 0.38	1.11 ± 0.41	0.425
**IRS-1[a] (pg/mL)**	1.62 ± 0.32	1.42 ± 0.27	**<0.001****

[a] Log 10 transformed. The analysis of variance (ANOVA) was adapted. Represented in means ± standard deviations. **Significant at $p<0.01$ threshold. Abbreviations: IGF-1 = insulin-like growth factor -1; IRS-1 = insulin receptor substrate.

Table S4. Differences in the metabolic signaling peptides between groups, adjusting for age, smoking and psychotropic medication use.

Metabolic Signalling peptides	AN (n=56)	RecAN (n=24)	HC (n=51)	*P*-value (Cohen's *d)*			
	Adjusted M ± SE			*Total Model*	AN vs. HC	AN vs. RecAN	RecAN vs. HC
**Leptin[a] (pg/mL)**	3.25 ± 0.09	3.66 ± 0.13	3.95 ± 0.10	**<0.001*** (0.88)**	**<0.001*** (0.93)**	**0.016* (0.58)**	0.076 (0.38)
**IGF-1 (pg/mL)**	5291.08 ± 194.61	5593.81 ± 281.3	5780.84 ± 205.17	0.258 (0.29)	0.100 (0.22)	0.378 (0.27)	0.593 (0.14)
**Insulin[a] (µIU/mL)**	1.086 ± 0.06	1.11 ± 0.08	1.06 ± 0.06	0.86 (0.09)	0.740 (0.06)	0.799 (0.00)	0.594 (0.14)
**IRS-1[a] (pg/mL)**	1.56 ± 0.04	1.55 ± 0.06	1.52 ± 0.05	0.798 (0.13)	0.510 (0.20)	0.880 (0.00)	0.680 (0.09)

[a] Log transformed; Significance threshold at *$p<0.05$, **$p<0.01$ or ***$p<0.001$; Significant *p*-values are presented in bold; Cohen *d* values of 0.2, 0.5 and 0.8 are considered to represent a small, moderate or large effect size, and represented in bold when effect size is moderate or large. Abbreviations: IGF-1 = insulin-like growth factor-1; IRS-1 = insulin receptor substrate-1; AN = anorexia nervosa; RecAN = recovered anorexia nervosa; HC = healthy controls; Adjusted M ± SE = adjusted mean ± standard errors; M ± SD = mean ± standard deviation.

Table S5. Differences in the metabolic signaling peptides between groups, adjusting for age, smoking and antidepressant use.

Metabolic signalling peptides	AN (n=56)	RecAN (n=24)	HC (n=51)	*P* value (Cohen *d)*			
	Adjusted M ± SE			*Total Model*	AN vs. HC	AN vs. RecAN	RecAN vs. HC
**Leptin[a] (pg/mL)**	3.26 ± 0.10	3.65 ± 0.13	3.95 ± 0.10	**<0.001*** (0.81)**	**<0.001*** (0.80)**	**0.020* (0.51)**	0.084 (0.48)
**IGF-1 (pg/mL)**	5271.97 ± 200.44	5602.43 ± 281.65	5797.77 ± 214.43	0.250 (0.30)	0.101 (0.21)	0.337 (0.25)	0.590 (0.23)
**Insulin[a] (µIU/mL)**	1.09 ± 0.06	1.11 ± 0.08	1.06 ± 0.06	0.847 (0.11)	0.727 (0.14)	0.786 (0.06)	0.569 (0.06)
**IRS-1[a] (pg/mL)**	1.55 ± 0.05	1.55 ± 0.06	1.53 ± 0.05	0.957 (0.06)	0.793 (0.13)	0.988 (0.00)	0.804 (0.00)

[a] Log transformed; Significance threshold at *$p<0.05$, **$p<0.01$ or ***$p<0.001$; Significant *p*-values are presented in bold; Cohen *d* values of 0.2, 0.5 and 0.8 are considered to represent a small, moderate or large effect size, and represented in bold when effect size is moderate or large. Abbreviations: IGF-1 = insulin-like growth factor-1; IRS-1 = insulin receptor substrate-1; AN = anorexia nervosa; RecAN = recovered anorexia nervosa; HC = healthy controls; Adjusted M ± SE = adjusted mean ± standard errors; M ± SD = mean ± standard deviation.

Table S6. Association between clinical and demographic characteristics with the metabolic signaling peptides in the anorexia nervosa group (n=56) controlling for age and smoking.

**Metabolic signalling peptides**	**Clinical and demographic characteristics**	***β***	***B*, 95% CIs**	***P* (*d*)**	***F (P)***
**Leptin[a] (pg/mL)**	***Body mass index***	0.44	0.24, 0.10; 0.38	**<0.001***** (**1.11**)	4.22 (**0.01***)
	Body fat %	0.53	0.07, 0.04; 0.11	**<0.001**** (**1.32**)	6.78 (**<0.001****)
	Depression diagnosis	0.14	0.2, -0.21; 0.61	0.337 (0.38)	0.41 (0.746)
**IGF-1 (pg/mL)**	***Body mass index***	0.15	157.37, -111.49; 426.23	0.246 (0.40)	4.70 (**0.006****)
	Body fat %	0.11	30.87, 38.66; 100.39	0.38 (0.33)	4.45 (**0.007****)
	Depression diagnosis	0.26	764.19, 52.38; 1476	**0.036* (0.66)**	6.04 (**0.001****)
**Insulin[a] (µIU/mL)**	***Body mass index***	0.20	0.06, -0.02; 0.14	0.139 (**0.52**)	0.89 (0.455)
	Body fat %	0.00	0.00, -0.02; 0.02	0.98 (0.11)	0.13 (0.942)
	Depression diagnosis	-0.07	-0.06, -0.29; 0.17	0.612 (-0.14)	0.22 (0.885)

**IRS-1[a] (pg/mL)**	***Body mass index***	-0.22	-0.06, -0.12; 0.01	0.09 (-0.46)	2.81 (**0.049***)
	Body fat %	-0.14	-0.01, -0.026; 0.01	0.28 (-0.29)	2.14 (0.106)
	Depression diagnosis	0.03	0.02, -0.16; 0.2	0.805 (0.17)	1.73 (0.172)

[a] Log transformed; Significance level at the threshold of *p<0.05, **p<0.01; Cohen d values of 0.2, 0.5 and 0.8 represents small, moderate, or large effects size. Significant p values and moderate and large effect sizes are presented in bold. $F(p)$ values represent the overall model statistics including all regressors while the $p(d)$ values refer to the individual regressor. Abbreviations: B = unstandardized coefficients; β = Standardized coefficients beta; CI = Confidence intervals; IGF-1 = insulin growth factor-1; IRS-1 = insulin receptor substrate-1.

Table S7. Association between eating disorder psychopathology and the metabolic signaling peptides in the anorexia nervosa group (n=56) controlling for age and smoking.

Metabolic Signalling peptides	Eating-related psychopathology	*β*	*B*, 95% CIs	*P* (*d*)	*F (p)*
Leptin[a] (pg/mL)	***Global score[a]***	0.09	0.55, -1.12; 2.22	0.513 (0.29)	0.24 (0.867)
	Eating restraint[a]	0.11	0.53, -0.83; 1.88	0.440 (0.32)	0.3 (0.826)
	Eating concern[a]	-0.01	-0.03, -1.42; 1.36	0.964 (-0.01)	0.1 (0.962)
	Shape concern[a]	0.07	0.39, -1.22; 2.00	0.630 (0.24)	0.18 (0.913)
	Weight concern[a]	0.18	0.78, -0.44; 1.99	0.205 (**0.50**)	0.65 (0.587)
IGF-1 (pg/mL)	***Global score[a]***	-0.01	-140.8, -3142.92; 2861.32	0.925 (-0.02)	4.13 (**0.011***)
	Eating restraint[a]	0.05	478.25, -1955.83; 2912.33	0.696 (0.2)	4.19 (**0.010***)
	Eating concern[a]	-0.07	-716.8, -3187.98; 1754.38	0.563 (-0.15)	4.27 (**0.009****)
	Shape concern[a]	-0.01	-156.6, -3043.63; 2730.42	0.914 (-0.03)	4.13 (**0.011***)
	Weight concern[a]	-0.02	-176.37, -2382.9; 2030.17	0.873 (-0.04)	4.14 (**0.011***)

**Insulin[a] (µIU/mL)**	***Global score[a]***	-0.17	-0.54, -1.46; 0.37	0.238 (-0.34)	0.61 (0.613)
	Eating restraint[a]	-0.18	-0.46, -1.2; 0.28	0.215 (-0.36)	0.66 (0.581)
	Eating concern[a]	-0.09	-0.24, -1.00; 0.52	0.528 (-0.18)	0.27 (0.850)
	Shape concern[a]	-0.11	-0.35, -1.23; 0.54	0.435 (-0.22)	0.34 (0.798)
	Weight concern[a]	-0.18	-0.42, -1.09; 0.25	0.21 (-0.360)	0.67 (0.574)
**IRS-1[a] (pg/mL)**	***Global score[a]***	-0.18	-0.48, -1.2; 0.24	0.19 (-0.36)	2.37 (0.082)
	Eating restraint[a]	-0.14	-0.32, -0.9; 0.27	0.286 (-0.29)	2.14 (0.107)
	Eating concern[a]	-0.09	-0.2, -0.81; 0.4	0.499 (-0.183)	1.88 (0.144)
	Shape concern[a]	-0.23	-0.6, -1.29; 0.08	0.083 (**-0.50**)	2.85 (**0.046***)
	Weight concern[a]	-0.15	-0.31, -0.84; 0.23	0.255 (-0.310)	2.20 (0.100)

[a] Log transformed; Significance level at the threshold of *p<0.05, ** p<0.01 or *** p<0.001; Cohen d values of 0.2, 0.5 and 0.8 represents small, moderate, or large effects size, and emboldened for moderate and large effect sizes. $F(p)$ values represent the overall model statistics including all regressors while the $p(d)$ values refer to the individual regressor. Abbreviations: EDE-Q = eating disorders examination questionnaire; IGF-1 = insulin growth factor-1; IRS-1 = insulin receptor substrate-1; B = unstandardized coefficients; β = standardized coefficients beta; CI = confidence intervals.

Table S8. Association between clinical and demographic characteristics and the metabolic signaling peptides in the acute and recovered anorexia nervosa group (n=80) controlling for age and smoking.

Metabolic signalling peptides	Clinical and demographic characteristics	*β*	*B*, 95% CIs	*P* (*d*)	*F (P)*
Leptin[a] (pg/mL)	*Body mass index*	0.37	0.1, 0.04; 0.15	<0.001** (0.92)	4.21 (0.008**)
	Body fat %	0.45	0.04, 0.02; 0.06	<0.001** (1.16)	6.80 (<0.001**)
	Depression diagnosis	0.06	0.09, -0.27; 0.45	0.636 (0.21)	0.28 (0.839)
IGF-1 (pg/mL)	*Body mass index*	0.15	72.92, -30.52; 176.35	0.164 (0.40)	5.39 (0.002**)
	Body fat %	0.18	31.99, -5.87; 69.85	0.096 (0.50)	5.73 (0.001**)
	Depression diagnosis	0.23	664.89, 61.64; 1268.14	0.031* (0.58)	6.51 (<0.001**)
Insulin[a] (µIU/mL)	*Body mass index*	0.18	0.03, -0.01; 0.06	0.118 (0.50)	1.90 (0.137)
	Body fat %	0.14	0.01, -0.01; 0.02	0.213 (0.39)	1.58 (0.201)
	Depression diagnosis	-0.02	-0.02, -0.22; 0.18	0.843 (-0.05)	1.04 (0.378)
	Body mass index	-0.08	-0.01, -0.04; 0.02	0.455 (-0.17)	2.36 (0.079)

**IRS-1[a] (pg/mL)**	***Body fat %***	-0.01	0.00, -0.01; 0.01	0.958 (-0.01)	2.15 (0.101)
	Depression diagnosis	0.03	0.02, -0.14; 0.17	0.811 (0.16)	2.17 (0.098)

[a] Log transformed; Significance level at the threshold of *p<0.05, **p<0.01 or ***p<0.001; Cohen d values of 0.2, 0.5 and 0.8 represents small, moderate, or large effects size, and are emboldened for moderate and large effect size. $F(p)$ values represent the overall model statistics including all regressors while the $p(d)$ values refer to the individual regressor. Abbreviations: IGF-1 = Insulin growth factor-1; IRS-1 = Insulin receptor substrate-1; B = unstandardized coefficients; β = standardized coefficients beta; CI = confidence intervals.

Table S9. Association between eating disorder psychopathology and the metabolic signaling peptides in the acute and recovered anorexia nervosa group (n=80) controlling for age and smoking.

Metabolic Signalling peptides	Eating-related psychopathology	*β*	*B*, 95% CIs	*P* (*d*)	*F (p)*
Leptin[a] (pg/mL)	***Global score[a]***	-0.24	-0.73, -1.43; -0.03	**0.043* (-0.50)**	1.64 (0.188)
	Eating restraint[a]	-0.21	-0.53, -1.11; 0.05	0.071 (-0.43)	1.33 (0.270)
	Eating concern[a]	-0.27	-0.77, -1.4; -0.15	**0.016* (-0.57)**	2.23 (0.091)
	Shape concern[a]	-0.21	-0.7, -1.45; 0.05	0.066 (-0.44)	1.38 (0.256)
	Weight concern[a]	-0.17	-0.5, -1.18; 0.18	0.144 (-0.35)	0.94 (0.426)
IGF-1 (pg/mL)	***Global score[a]***	-0.10	-559.84, -1796.44; 676.75	0.370 (-0.20)	4.93 (**0.003****)
	Eating restraint[a]	-0.11	-538.23, -1548.84; 472.39	0.292 (-0.23)	5.05 (**0.003****)
	Eating concern[a]	-0.11	-552.43, -1670.3; 565.45	0.328 (-0.21)	4.99 (**0.003****)
	Shape concern[a]	-0.11	-683.06, -1998.87; 632.75	0.304 (-0.22)	5.03 (**0.003****)
	Weight concern[a]	-0.05	-281.33, -1467.82; 905.16	0.638 (-0.10)	4.70 (**0.005****)

**Insulin[a] (µIU/mL)**	***Global score[a]***	-0.14	-0.24, -0.64; 0.16	0.236 (-0.28)	1.53 (0.214)
	Eating restraint[a]	-0.16	-0.24, -0.56; 0.09	0.151 (-0.33)	1.76 (0.162)
	Eating concern[a]	-0.10	-0.16, -0.52; 0.21	0.391 (-0.20)	1.29 (0.284)
	Shape concern[a]	-0.09	-0.18, -0.6; 0.25	0.416 (-0.19)	1.26 (0.293)
	Weight concern[a]	-0.17	-0.29, -0.67; 0.09	0.136 (-0.35)	1.82 (0.151)
**IRS-1[a] (pg/mL)**	***Global score[a]***	-0.08	-0.11, -0.42; 0.2	0.470 (-0.17)	2.34 (0.08)
	Eating restraint[a]	-0.07	-0.08, -0.33; 0.17	0.539 (-0.14)	2.29 (0.085)
	Eating concern[a]	-0.03	-0.04, -0.31; 0.24	0.798 (-0.06)	2.18 (0.098)
	Shape concern[a]	-0.12	-0.18, -0.51; 0.15	0.273 (-0.25)	2.59 (0.059)
	Weight concern[a]	-0.10	-0.13, -0.42; 0.17	0.395 (-0.20)	2.42 (0.073)

[a] Log transformed; Significance level at the threshold of *p<0.05, **p<0.01 or **p<0.001; d values of 0.2, 0.5 and 0.8 represents small, moderate, or large effects size, and are emboldened for moderate and large effect size. F(p) values represent the overall model statistics including all regressors while the p(d) values refer to the individual regressor. Abbreviations: EDE-Q = eating disorders examination questionnaire; IGF-1 = insulin growth factor-1; IRS-1 = insulin receptor substrate-1; B = unstandardized coefficients; β = standardized coefficients beta; CI = confidence intervals.

2. Supplementary figures

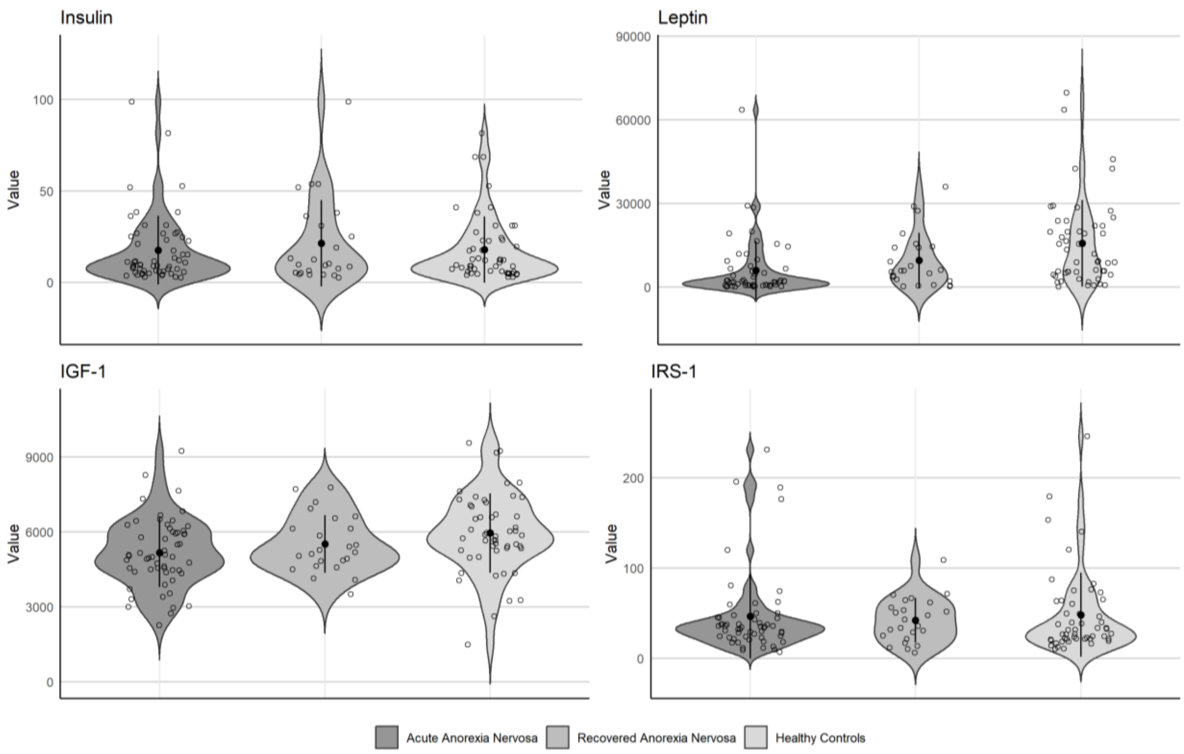

Figure S1. Violin plot of metabolic signaling peptides divided by study group.
Abbreviations: IGF = insulin-like growth factor-1; and IRS-1 = insulin receptor substrate-1.
The plot represents the data distribution of leptin, insulin, IGF-1 and IRS-1. Leptin, IGF-1 and IRS-1 were measured in pg/mL, while insulin was measured in µIU/mL.
